# Case report: failure under azithromycin treatment in a case of bacteremia due to *Salmonella enterica* Paratyphi A

**DOI:** 10.1186/1471-2334-14-404

**Published:** 2014-07-20

**Authors:** Tetsuro Kobayashi, Kayoko Hayakawa, Momoko Mawatari, Kazuhisa Mezaki, Nozomi Takeshita, Satoshi Kutsuna, Yoshihiro Fujiya, Shuzo Kanagawa, Norio Ohmagari, Yasuyuki Kato, Masatomo Morita

**Affiliations:** 1 Disease Control and Prevention Center, National Center for Global Health and Medicine, Shinjuku-ku Toyama 1-21-1, 162-8655 Tokyo, Japan; 2 Microbiology Laboratory, National Center for Global Health and Medicine, Shinjuku-ku Toyama 1-21-1, 162-8655 Tokyo, Japan; 3 National Institute of Infectious Diseases, Shinjuku-ku Toyama 1-23-1, Tokyo, Japan

**Keywords:** *Salmonella* Paratyphi, Enteric fever, Azithromycin, Treatment failure, Returned traveler

## Abstract

**Background:**

Limited information is available regarding the clinical efficacy of azithromycin for the treatment of enteric fever due to fluoroquinolone-resistant *Salmonella* Typhi and *Salmonella* Paratyphi among travelers returning to their home countries.

**Case presentation:**

We report a case of a 52-year-old Japanese man who returned from India, who developed a fever of 39°C with no accompanying symptoms 10 days after returning to Japan from a 1-month business trip to Delhi, India. His blood culture results were positive for *Salmonella* Paratyphi A. He was treated with 14 days of ceftriaxone, after which he remained afebrile for 18 days before his body temperature again rose to 39°C with no apparent symptoms. He was then empirically given 500 mg of azithromycin, but experienced clinical and microbiological failure of azithromycin treatment for enteric fever due to *Salmonella* Paratyphi A. However, the minimum inhibitory concentration (MIC) of azithromycin was not elevated (8 mg/L). He was again given ceftriaxone for 14 days with no signs of recurrence during the follow-up.

**Conclusion:**

There are limited data available for the treatment of enteric fever using azithromycin in travelers from developed countries who are not immune to the disease, and thus, careful follow-up is necessary. In our case, the low azithromycin dose might have contributed the treatment failure. Additional clinical data are needed to determine the rate of success, MIC, and contributing factors for success and/or failure of azithromycin treatment for both *Salmonella* Typhi and *Salmonella* Paratyphi infections.

## Background

*Salmonella enterica* Typhi and *Salmonella enterica* Paratyphi A, B, and C are the causative pathogens of enteric fever in tropical and subtropical countries such as southern and southeast Asia and Africa [1], and fluoroquinolone-resistant strains of *Salmonella* Typhi and *Salmonella* Paratyphi A have recently emerged in these countries [2]. In developed countries, these strains cause enteric fever in travelers returning from the endemic areas [3]. Azithromycin and the third-generation cephalosporins (e.g., ceftriaxone) are the drugs of choice for treatment of fluoroquinolone-resistant *Salmonella* Typhi and *Salmonella* Paratyphi [4]. However, the azithromycin breakpoints for *Salmonella* Typhi and *Salmonella* Paratyphi A have not been defined by the Clinical and Laboratory Standard Institutions (CLSI) or the European Committee on Antimicrobial Susceptibility Testing (EUCAST) criteria [5,6]. The number of studies regarding the clinical efficacy of azithromycin for the treatment of enteric fever is still limited, and to our knowledge, all of them were conducted in endemic areas [7-11]. Here, we report a case of azithromycin treatment failure in a Japanese man with *Salmonella* Paratyphi A infection who returned from travel.

## Case presentation

A 52-year-old Japanese man developed a fever of 39°C with no accompanying symptoms 10 days after returning to Japan from a 1-month business trip to Delhi, India. He presented to a local clinic 5 days after the fever developed. Blood cultures grew with Gram-negative rods (GNR) after 3 days, which were subsequently identified as *Salmonella* Paratyphi A with unknown antibiotic susceptibility. The patient was transferred to our hospital to complete his 14-d therapy with ceftriaxone (2 g daily). He was afebrile for 18 days following the completion of the antibiotic therapy, after which his body temperature rose to 39°C with no apparent symptoms. He presented to our hospital on Day 3 of the second fever. A blood culture was performed, and the patient was empirically treated with oral azithromycin (500 mg daily) owing to a suspected relapse of enteric fever. The patient was compliant with the oral antibiotic regimen. As the blood culture results showed GNR growth in 2 days, he was readmitted to our hospital for further treatment. We continued azithromycin treatment under direct observation by the medical staff for a total of 7 days. GNR was again identified as *Salmonella* Paratyphi A, which was found to be susceptible to ceftriaxone (minimum inhibitory concentration [MIC], 0.12 mg/L), resistant to nalidixic acid, and showed decreased susceptibility to ciprofloxacin (MIC, 0.5 mg/L). The MIC’s for azithromycin were 8 mg/L and 16 mg/L, as determined in a reference laboratory using the E-test (Biomerieux Co. Ltd., Tokyo, Japan) and broth micro dilution (BMD), respectively.

Despite the uninterrupted 7 days of oral azithromycin treatment under direct observation, the patient remained febrile until the final day of the regimen. Another blood culture was performed, and the treatment was switched to ceftriaxone because of suspected azithromycin treatment failure. The blood culture showed GNR growth, which was again identified as *Salmonella* Paratyphi A with the same MICs for ceftriaxone, ciprofloxacin, and azithromycin as the initial culture results. Fluorodeoxyglucose positron emission tomography (FDG- PET) showed slightly thickened intestinal wall of the colon. However, no other inflammatory focuses suggesting deep-seeded infection, such as abscesses and aortitis, were detected. The abdominal ultrasound showed no intra-abdominal abscess on the thickened intestinal wall. The patient defervesced on Day 3 of ceftriaxone, and the result of the follow-up blood culture performed on Day 4 of ceftriaxone therapy yielded no growth. After completion of 14 days of antibiotic treatment, the patient was discharged with no signs of recurrence during the follow-up.

### Discussion

Enteric fever due to nalidixic acid-resistant *Salmonella* Typhi and/or *Salmonella* Paratyphi A is generally treated with ceftriaxone or azithromycin [12,13] with previous reports demonstrating the efficacy of azithromycin in the treatment of enteric fever [7-9]. Moreover, the probability of recurrence is < 3% when treated with azithromycin compared to 3–6% when treated with ceftriaxone. Therefore, treatment with azithromycin may be preferred for quinolone-resistant *Salmonella* Typhi and *Salmonella* Paratyphi A [8,14]. However, data are currently only available for endemic regions of enteric fever where immunity to the disease may contribute to the high success rates.

To the best of our knowledge, there has only been fewcase reports of treatment failure with azithromycin for *Salmonella* Paratyphi A. In one report, the MIC for azithromycin was > 64 mg/L, which was assumed to be azithromycin-resistant; however, the exact mechanism of resistance has not been defined [15]. In another report, a case of recurrent, multifocal Salmonella enterica serotype Paratyphi A breast abscesses was presented which relapsed despite surgery and multiple courses of antibiotics, including co-trimoxazole and azithromycin [16]. Although the bioavailability of oral azithromycin was reported to be approximately 37%, which is lower than that for fluoroquinolones, it has been suggested that the intracellular concentration of azithromycin is 50–100 times greater than that in serum and that the bactericidal effect remains stable regardless of a high MIC [17,18]. The recent CLSI guidelines and the EUCAST criteria do not specify the MIC for azithromycin when treating *Salmonella* Typhi or *Salmonella* Paratyphi A [5,6]. However, the EUCAST states that the wild-type isolates of *Salmonella* Typhi have an MIC ≤ 16 mg/L [6]. In a study conducted in India, the MIC distributions for *Salmonella* Typhi and *Salmonella* Paratyphi A ranged from 0.0612 to 64 mg/L and from 1 to 32 mg/L, respectively, and the MIC90 values were 24 mg/L for both serovars [19]. Recent report on MIC distribution of typhoidal Salmonella isolates of returned travelers showed that azithromycin MICs were 2–256 μg/mL among the 354 isolates, and a minority of the S. Paratyphi A isolates showed an MIC ≤ 8 mg/L [20].

In the present case, we experienced clinical and microbiological treatment failure with azithromycin for enteric fever due to *Salmonella* Paratyphi A despite the fact that the MIC for azithromycin was not elevated (8 mg/L by E-test and 16 mg/L by BMD). Previous studies evaluated the efficacy of azithromycin therapy used various regimens as summarized in Table 1. Most of the dosages used ranged from 10 mg/kg to 20 mg/kg. A review published in 2005 recommends 8–10 mg · kg^-1^ · dose^-1^ azithromycin for enteric fever caused by either *Salmonella* Typhi or *Salmonella* Paratyphi A [12]. Owing to the patient’s weight (80 kg) at the time of admission, 500 mg of azithromycin [13], which was 6.25 mg/kg, might have been an inadequate dose when compared to previously recommended doses. Further studies are necessary in terms of optimal route of administration, and appropriate dose and duration especially among returned travelers who are not immune to typhoid.

**Table 1:** **Reported efficacy of azithromycin for treatment of *Salmonella *** **Typhi and *Salmonella *** **Paratyphi**

**Author, year**	**Country**	**Patients population**	**Azithromycin dose**	**Body weights of patients (kg)**	**Route of administration**	**Efficacy**	MIC of isolates, g/L*	**References**
Girgis, 1999	Egypt	n = 36, mean age (range): 19.6 (18–30)	1 g/day on the first day, followed by 500 mg/day on the next 6 days	NA	Oral	Clinical cure rate: 100%	BMD, MIC*_90_ (range): 8 (4–16)	[9]
Butler, 1999	India	n = 42, mean age (range): 26.2 (17–53)	500 mg/day for 7 days	Mean 52.2 (range: 40–74)	Oral	Clinical cure rate: 88% (within 8 days), 100% (within 14 days)	BMD MIC*_90_ (range): 16 (4–32)	[7]
Chinh, 2000	Vietnam	n = 44, mean age (range): 26.6 (15–68)	1 g/day for 5 days	Mean 47.3 (range: 34–60)	Oral	Clinical cure rate: 95.5%	E-test, MIC_90_ (range): 8 (4–16)	[10]
Parry, 2007	Vietnam	n = 62, mean age (range): 10.5 (4–42)	10 mg/kg/day for 7 days	Mean 24 (range: 12–58)	Oral	Clinical cure rate: 82%	E-test MIC_90_ (range): 16 (4–32)	[8]
Dolecek, 2008	Vietnam	n = 142, median age 11 yrs, (range 1–41)	20 mg/kg/day for 7 days	Median 24.5 (9.5–57)	Oral	Treatement failure: 9.3%	E-test, MIC_90_ (range): 12 (4–16)	[11]

Abbreviations: *BMD* broth microdilution, *MIC* Minimum inhibitory concentrations, *NA* data not available.

*MIC_90_ were determined based on the data provided in refeneces.

In this case, no infectious focus such as arteritis or abscesses was detected by the PET-CT scan and abdominal sonography. We suspected that the thickening of intestinal wall of the colon might be due to the hypertrophy of Peyer’s patches; one of the classic presentations observed in cases of enteric fever due to *Salmonella* Typhi or *Salmonella* Paratyphi [21].

## Conclusions

There are limited data available for the treatment of enteric fever using azithromycin in travelers from developed countries who are not immune to the disease, and thus, careful follow-up is necessary. Additional clinical data are needed to determine the rate of success, MIC, and contributing factors for success and/or failure of azithromycin treatment for both *Salmonella* Typhi and *Salmonella* Paratyphi.

## Consent

Written informed consent was obtained from the patient for the publication of this case report. A copy of the written consent is available for review by the Editor of this journal.

## Competing interests

The authors declare that they have no competing interests.

## Authors’ contributions

TK was responsible for drafting the manuscript, and clinical management of the case. KH and YK supervised the work and helped to draft the manuscript. KM participated in the microbiological work-ups. MM, NT, SK, YF, SK, and NO were responsible for clinical management of the case. All authors have read the manuscript and accepted the final version.

## Pre-publication history

The pre-publication history for this paper can be accessed here:

http://www.biomedcentral.com/1471-2334/14/404/prepub
